# Alcohol intoxication and mental health among adolescents – a population review of 8983 young people, 13–19 years in North-Trøndelag, Norway: the Young-HUNT Study

**DOI:** 10.1186/1753-2000-3-18

**Published:** 2009-06-23

**Authors:** Arve Strandheim, Turid Lingaas Holmen, Lindsey Coombes, Niels Bentzen

**Affiliations:** 1 Norwegian University of Science and Technology, the Faculty of Medicine, Department of Public Health and General Practice, Trondheim, Norway; 2 Norwegian University of Science and Technology, the Faculty of Medicine, Department of Public Health and General Practice, HUNT research centre, Verdal, Norway; 3 Nord-Trøndelag Health Trust, Sykehuset Levanger, Levanger, Norway; 4 Oxford Brookes University, the School of Health and Social Care, Oxford, UK

## Abstract

**Background:**

The aims of this study were to describe alcohol use among Norwegian teenagers and investigate the associations between mental health problems and alcohol intoxications with focus on age and gender.

**Methods:**

Population based, cross-sectional survey addressing all adolescents aged 13–19 years, attending secondary or high school in North – Trøndelag County, Norway. 8983 youths (91%) answered the Young-HUNT questionnaire in the 1995–1997 survey. Logistic regression models were used to study associations.

**Results:**

80% of the respondents reported that they had tried drinking alcohol, and 57% had been intoxicated at least once. The proportion of the students, which had tried alcohol, was equal in both genders and increased with age. Attention problems and conduct problems were strongly associated with frequent alcohol intoxications in both genders. Anxiety and depressive symptoms among girls were also related to high numbers of intoxications

**Conclusion:**

Gender differences in number of alcohol intoxications were small. There was a close association between both conduct and attention problems and high alcohol consumption in both genders. Girls with symptoms of anxiety and depression reported more frequent alcohol intoxications.

## Background

In Europe earlier alcohol debut, higher alcohol consumption, and more polysubstance use among adolescents have been documented in the last decade [1-4]. Many studies, especially from the US emphases the health risks from early alcohol debut [5,6]. Alcohol intoxication has been described as a particular risk in adolescence, both from experiments and epidemiological research [7,8]. Adolescents in Europe, Australia and the US were attributed a pattern of binge drinking with frequent alcohol intoxication in several studies [2,7-9].

Associations of alcohol use in adolescence with several mental health problems, such as conduct problems, hyperactivity/attention problems, depression, anxiety and suicidal behaviour has been reported in several studies [2,10-14]. Childhood hyperactivity and attention deficit is considered a risk factor for later alcohol- and substance misuse[15,16], although some researchers explain this as mediated by deviant peer affiliation [17]. On the other hand problem behaviours as conduct problems, alcohol and drug use have been considered a common syndrome in several studies [18,19].

Age and gender specific population studies are rare, even though important differences are indicated in several articles [20-22]. Mood and anxiety disorders are overrepresented among women, while hyperactivity-, conduct- and substance use disorders are overrepresented among men, at least in clinical samples [23,24]. Existing population based research also indicates important gender- and age differences in the prevalence of common adolescent mental health problems [24-26], with possible influence on the association with alcohol use. Incidence rates indicates early onset (age 18–24) of alcohol and substance use disorders [25], with the closest relationship between alcohol use and mental health problems among the youngest adolescents [27]. Few European and especially Scandinavian population data are describing adolescents' alcohol use and relation to mental health [12]. The need for further studies has been identified in recent papers [28,29] who pointed out that research in this area often have used small clinical samples. The Young-HUNT data set includes mental health problems in the total population of a region, which enables association studies with alcohol use and lifestyle data.

Thus the aims of this study were to examine the relationships between alcohol intoxications and common mental health problems in a total adolescence population, and focus on possible gender and age differences.

## Methods

### Study population and design

The county of Nord-Trøndelag, situated in the central Norway, has about 127.000 inhabitants. The Nord-Trøndelag Health Study (HUNT), conducted in 1995/97, invited all inhabitants 13 years and older to the survey. Students in secondary school and high school, 13–19 years, participated in the youth part of the study; Young-HUNT1, used in this work. Norway has a public school system for all students regardless of handicap or learning difficulties, special schools are rare. Secondary school is compulsory, while students have the right to attend a free high school education. Of a total of 10202 adolescents only 285 were not attending school. The design is cross-sectional, exploring associations of mental health problems and alcohol use in adolescence 13 – 19 years of age. The total population was divided in gender and age groups.

### Measures

The students completed a comprehensive self-administered questionnaire in an exam setting during a school hour. Questions on physical and mental health, life style and socio-demographic factors were included.

To define problem groups from different scales, scores above the 70th percentile was used systematically in accordance to previous studies of anxiety and depression [30].

### Variables

#### Alcohol use

Self reported numbers of lifetime alcohol intoxication episodes was the primary outcome measure. More than 10 intoxications were defined as high alcohol use in all age groups. The same definition has been used in previous studies and is known to be easy to collect, fairly stable and easily remembered by the youth [31]. In the descriptive part of the analysis, number of alcohol intoxications were divided in three; "no reported intoxication", "one to 10 intoxications" or "more than 10 intoxications". This gave substantial counts in all age and gender groups, and was supposed to discriminate different levels of alcohol involvement and risk. For the binary logistic regression analysis, the data regarding intoxication episodes were dichotomized into one group with 0–10 intoxication episodes, the other group with more than 10 intoxications [31].

To gain other measures of alcohol use the students also were asked to report the amount of beer, wine or spirit they usually drank during a 2-week period. The data were recalculated to grams of alcohol and then to alcohol units (8 g pr unit) [32]. The students drinking above the 70th percentile for this population, 3 or more units in 14 days, was defined high volume drinkers in the age group 13–19 years [33].

#### Mental health

The mental health variables were derived from the mental health "Symptom Check List" and the school adjustment-part of the questionnaire.

#### Anxiety and depression

Symptoms of anxiety and depression were measured by SCL-5 (Symptom Check List), a five-item scale based on SCL-25, proven reliable in previous studies [30,34]. A factor analysis gave only one factor with Eigenvalue > 1, making distinction between anxiety and depressive symptoms impossible. According to this analysis all five items were included in the anxiety/depressive variable (Anxiety problems: "been constantly scared and uneasy", "felt tense and restless" and "worries too much about different matters". Depressive problems: "felt hopeless when thinking of future" and "felt down or sad"). All questions had four alternative responses ranging from one: "not at all" to four: "extremely". The aggregated score of the SCL-5 questions were summarized and ranged from five to 20. These scores constitute no true interval scale and was recalculated into dichotomous categorical variables with more than 8 points as the cut off point, according to former studies [30,34].

#### Attention and conduct problems

The school adjustment part of the questionnaire with totally 14 items used to define behavioral problems, has been described previously [35]. A factor analysis using Varimax rotation with Kaiser Normalization defined 2 independent factors: attention- and conduct problems, both with an Eigenvalue > 1. Attention problems include the items how often "do you have trouble concentrating in class" and "don't manage to be calm in class". Conduct problems include how often do you "quarrels with the teacher", "get into fights" and "get scolded by the teacher". All items had four alternative responses from one: "never" to four: "very often". Each category was recalculated into aggregated scores. Attention problems had a distribution from two to 8, with median at four. Conduct problems ranked from tree to 12. Both scales were dichotomised into "high" or "low" problem score, defining a high problem group over the 70^th ^percentile [30]. Scores of five or more constitutes the high problem groups concerning both attention and conduct problems.

### Analysis

Descriptive statistic was derived from contingency tables with use of Pearson Chi-Square tests. In logistic regression models alcohol intoxication was used as dependent variable. Independent variables included were, anxiety/depression, conduct problems and attention problems. Odds ratios (OR) and their corresponding 95% confidence intervals (CI) were calculated from all analysis. Since alcohol consumption is known to increase with age and may differ between genders, interactions were calculated for each independent variable. Significant interaction was found for anxiety/depressive symptoms by gender (p = 0.001), and nearly significant for anxiety/depressive symptoms by age (p = 0.079). These interactions are both statistical and clinical meaningful, consequently all models were stratified by gender and the two age groups 13 – 5 and 16 – 19 years.

### Ethical considerations

Each student signed a written consent form. For the students younger than 16 the parent also gave their written consent. Anonymous results were communicated to local health authorities, to be used for planning of health services, in accordance with the consent given. The study was evaluated and approved by the Regional Medicine Ethics Committee and the Norwegian Data Inspectorate Board.

## Results

### Frequencies of alcohol use and mental health problems

Among the 9917 students invited in the target grades, 91% (totally 8984, 4507 boys, 4424 girls) answered the questionnaire, 94.8 in secondary schools and 85.6 in high schools. Of all the youths who completed the questionnaire, 43.3% reported that they never had been intoxicated by alcohol, and 28.9% reported more than 10 episodes of intoxication. The number of intoxications increased with age both among boys and girls (table 1). Gender difference was not found among those without experience of intoxication. Only slightly more boys than girls reported more than 10 intoxications. The gender difference was more obvious in the high volume-drinking group, which included 28.9% of boys and 19.6% of girls.

**Table 1:** Number of alcohol intoxications and high volume drinking by gender and age in Nord-Trøndelag, Norway 1995–97.

		Boys				Girls			
		Age 13–15		Age 16–19		Age 13–15		Age 16–19	
		N	%	N	%	N	%	N	%

Intoxication	0	1575	68.2	400	18.2	1547	66.5	352	16.6
	1–10	584	25.3	563	25.6	640	27.5	699	33.0
	>10	149	6.5	1242	56.2	138	5.9	1066	50.4
									
									
Alcohol volume*	low	2045	88.9	1143	52.4	2151	92.8	1405	66.7
	High	256	11.1	1039	47.6	168	7.2	700	33.3

* Low is defined as less than 3 alcohol units pr 14 days, high as 3 or more units.

Attention problems, anxiety and depressive symptoms increased with age, and were more common in girls compared to boys; while high conduct problems decreased with age and were more common in boys (table 2).

**Table 2:** Mental health problems by gender and age in adolescents, Nord-Trøndelag, Norway 1995–97

	Boys				Girls			
	
	Age 13–15		Age 16–19		Age 13–15		Age 16–19	
	N	%	N	%	N	%	N	%
High attention problems score	499	22.7	580	27.1	551	24.5	614	29.6
High conduct problems score	964	43.7	744	34.9	673	29.9	461	22.2
High combined anxiety/depressive symptoms score*	295	13.3	400	18.9	566	25.2	769	37.6

* Anxiety symptoms and depressive symptoms derived from SCL-5, presented as a common variable based on all the 5 questions in SCL-5.

### Associations of alcohol intoxication and mental health

Attention problems, conduct problems, anxiety and depressive symptoms were highly associated with the number of alcohol intoxications the students had experienced. In the total population 25.1 – 26.8% of the student in the low problem groups had experienced more than 10 intoxications; while the corresponding prevalences in the high symptom score groups were 35.5 – 42.6% 8 (p = 0.001 for all) (table 3).

**Table 3:** Prevalence of alcohol intoxications by mental health problems among adolescents, Nord-Trøndelag, Norway 1995–97

			Not intoxicated	1–10 intoxications	>10 intoxications
Attention problems	Low	N	3026	1783	1614
		%	47.1	27.8	25.1
	High	N	618	673	955
		%	27.5	30.0	42.6
					
Conduct problems	Low	N	2684	1584	1561
		%	46.0	27.2	26.8
	High	N	966	868	1008
		%	34.0	30.5	35.5
					
Anxiety and depressive symptoms	Low	N	3039	1814	1740
		%	46.1	27.5	26.4
	High	N	624	623	783
		%	30.7	30.7	38.6

P = 0.0001 for the differences between high and low symptom groups on more than 10 intoxications estimated by Pearsons Chi-square/likelihood ratio

According to the interactions shown, the material was stratified in groups, with respect to gender and age (13–15, 16–19). Stratified analysis showed that conduct problems were the most important association with boys' intoxications, followed by attention problems (table 4). The importance of both conduct (OR 3.5 versus 2.1) and attention problems (OR 3.0 versus 1.7) on the number of intoxications diminished with age. Among girls, the association of attention- and conduct problems with alcohol intoxications were equally strong, both diminishing with age. The changes by age of attention problems among boys seemed robust with separated Confidence Intervals, while the age changes among the other factor were questionable due to overlapping confidence intervals. Combined anxiety and depressive symptoms was associated with higher intoxication frequency only in girls.

**Table 4:** Odds Ratio of >10 alcohol intoxications with high score of mental health problems. Binary logistic regression stratified by age and gender. Nord-Trøndelag, Norway 1995–97

			OR	95% CI	p-value
Boys	13–15				
		High attention problems	3.0	2.1–4.4	0.0001
		High conduct problems	3.5	2.3–5.3	0.0001
		High anxiety/depr. symp.	0.9	0.5–1.5	0.617
					
	16–19				
		High attention problems	1.7	1.3–2.1	0.0001
		High conduct problems	2.1	1.7–2.6	0.0001
		High anxiety/depr. symp.	1.0	0.8–1.2	0.8
					
Girls					
	13–15				
		High attention problems	2.7	1.8–4.0	0.0001
		High conduct problems	2.4	1.6–3.6	0.0001
		High anxiety/depr. symp.	1.7	1.1–2.5	0.015
					
	16–19				
		High attention problems	2.1	1.7–2.0	0.0001
		High conduct problems	1.9	1.5–2.4	0.0001
		High anxiety/depr. symp.	1.4	1.1–1.7	0.001

## Discussion

### Summary of main findings

Alcohol use is prevalent among students aged 13 – 19 years in North-Trøndelag, Norway, both measured by volume and frequency of intoxications. The high numbers of alcohol intoxication compared to the relative low total amount of alcohol consumed, indicated a pattern of heavy episodic drinking among adolescence in Nord-Trøndelag. The gender difference in alcohol use was small, consistent with description of diminishing gender differences both from Europe, US and Australia in the last decade [1,36-38]. There was a close association of high alcohol consumption with conduct and attention problems in both genders. Among boys, conduct problems had the closest association to frequent intoxications, while girls with attention problems and conduct problems where equally exposed. The strong relationship between frequent alcohol intoxication and symptoms of attention and conduct problems, has also been described in clinical research [39-41]. Few epidemiological studies deal with the relationship of individual mental health factors and possibly dangerous alcohol consumption [27,39]. In the Young HUNT data depressive- and anxiety symptoms were associated with high number of intoxications in girls, but not in boys. Some previous works find interaction with gender, and important gender differences [20], while others do not [27]. The divergence in findings may be due to methodological differences or local variation.

A substantial amount of literature is focused on the effect of early alcohol debut on mental health [42-46]. This study emphases the co-occurrence of mental health problems and intoxicant alcohol behaviour, consistent with recent clinical reports [47-49].

### Limitations and strengths

The current data set is cross-sectional, and not suitable to determine causal relations. However, the Young-HUNT is a total cohort study, with a high response rate, and many methodological problems concerning samples could be excluded. 91% of the student participated; most of the nonparticipants were absent from school for health reasons, some did not consent and very few forms were not readable. The response rate from the adolescents not in school or in vocational training was low, and were excluded from the study. Even if they were few they would be an interesting group to include, and could possibly contribute to the associations measured.

The data is collected from adolescence 13–19 years old in the phase were both alcohol habits and mental health profile emerges. The questionnaire was designed with some pre-validated variables like SCL-5, while the questions of school behaviour have few earlier evaluations. These questions are similar to core diagnostic questions in DSM IV, but neither the questions in the school part of Young-HUNT, nor the SCL-5 questions have diagnostic specificity, and can only be used as measures of problems in the clusters addressed. All information used in the study was self-reported, and can contain both over- and under-estimates. Some recent studies indicated that self-report on alcohol are reasonably reliable for adolescents [31,50].

### Clinical and scientific implications

The Young-HUNT study may suggest that adolescents with attention- and conduct problems are at high risk for alcohol problems. This is in accordance with recent research on attention and hyperactivity disorders described as precursor of alcohol and other drug misuse [17,51-54]. Conduct problems, however, is known to be related to adolescents' alcohol use, and the problems tends to develop further in young adulthood [18,55]. Alcohol intoxication in adolescence may indicate an independent risk for later mental health problems [56]. In accordance with the present findings and substantial international research, adolescents with attention- and conduct problems and their families, should be offered information and health advice adjusted to their situation. The possibility of a complicating alcohol habit to their previous behavioural problems is probable. Caretakers and doctors that handle girls with anxiety and depressive symptoms should bear in mind that this may increase the risk for alcohol problems developing in adolescence. All adolescents with conduct – and attention problems and girls with anxiety and depressive symptoms might be a very relevant target for indicated alcohol prevention interventions. On the other hand, the findings from this study do not oppose universal preventive strategies (i.e. age limits, sales restrictions); it might benefit the whole adolescence population, and give extra health promotion also for the groups at risk.

The co-occurrence of frequent alcohol intoxications and psychiatric problems in adolescence identified in this study may indicate common background traits or possible existence of causal relations between mental health and alcohol use.

This study supports the view that attention problems and conduct problems may play a part in the development of harmful alcohol use in adolescence. Another interpretation is that attention-, conduct- and substance use problems are different expression of the same traits in the adolescence population [57]. That alcohol intoxication or alcohol use should generate the mental heath problems described, seems less likely, especially conduct- and attention problem tend to emerge early in childhood. This cross-sectional material does not solve that problem, but further prospective studies and qualitative research might contribute.

## Conclusion

Gender differences in number of alcohol intoxications among adolescence 13–19 years old in Nord-Trøndelag, Norway were small. The number of intoxications increased with age both among boys and girls. There was a close association between both conduct and attention problems and frequent alcohol intoxications in both genders. Girls with high scores of depressive and anxiety symptoms reported more frequent alcohol intoxications compared to girls with few symptoms. The associations described were strongest in the youngest groups (13–16). Both clinical practice and prevention may emphasis more on the alcohol habits of young adolescents with attention- and conduct problems, and girls with anxiety or depressive symptoms. Further prospective or qualitative studies may contribute to the understanding of alcohol- and mental health problems developing in adolescence.

## Competing interests

The authors declare that they have no competing interests.

## Authors' contributions

AS participated in designing the study, performed the analysis and drafted the article. TLH is the PI of the Young-HUNT-study and participated in the design, statistical analysis, drafting and presentation of the results. LC participated in the description of background, especially UK and European research, statistical analysis, drafting and presentation. NB was essential in the development of the idea, description and thinking of the study, as well as drafting of the article. All authors have read and approved of the final manuscript.
